# Ensnaring membrane type 1-matrix metalloproteinase (MT1-MMP) with tissue inhibitor of metalloproteinase (TIMP)-2 using the haemopexin domain of the protease as a carrier: a targeted approach in cancer inhibition

**DOI:** 10.18632/oncotarget.15165

**Published:** 2017-02-07

**Authors:** Bingjie Jiang, Yan Zhang, Jian Liu, Anastasia Tsigkou, Magdalini Rapti, Meng Huee Lee

**Affiliations:** ^1^ Department of Biological Sciences, Xian Jiaotong Liverpool University, Suzhou 215123, China; ^2^ Cancer Research UK Cambridge Institute, University of Cambridge, Robinson Way, Cambridge CB2 0RE, United Kingdom

**Keywords:** MT1-MMP, TIMP, cancer, haemopexin, protein engineering

## Abstract

Metastatic cancer cells express Membrane Type 1-Matrix Metalloproteinase (MT1-MMP) to degrade the extracellular matrix in order to facilitate migration and proliferation. Tissue Inhibitor of Metalloproteinase (TIMP)-2 is the endogenous inhibitor of the MMP. Here, we describe a novel and highly effective fusion strategy to enhance the delivery of TIMP-2 to MT1-MMP. We can reveal that TIMP-2 fused to the haemopexin +/− transmembrane domains of MT1-MMP (two chimeras named T2^PEX+TM^ and T2^PEX^) are able to interact with MT1-MMP on the cell surface as well as intracellularly. In the case of T2^PEX+TM^, there is even a clear sign of MT1-MMP:T2^PEX+TM^ aggregation by the side of the nucleus to form aggresomes. *In vitro*, T2^PEX+TM^ and T2^PEX^ suppress the gelatinolytic and invasive abilities of cervical carcinoma (HeLa) and HT1080 fibrosarcoma cancer cells significantly better than wild type TIMP-2. In mouse xenograft, we further demonstrate that T2^PEX^ diminishes cervical carcinoma growth by 85% relative to the control. Collectively, our findings indicate the effectiveness of the fusion strategy as a potential targeted approach in cancer inhibition.

## INTRODUCTION

Membrane Type 1-Matrix Metalloproteinase (MT1-MMP, also known as MMP-14) is a zinc-containing endoproteinase of the Metzincin superfamily that counts among its members the matrix metalloproteinases (MMPs), the membrane-anchored adamalysins (ADAMs) as well as the soluble ADAMTS proteases with a characteristic type I thrombospondin motifs at the C-terminal ends [1–5]. Of the twenty five MMPs identified to date, MT1-MMP is by far the most prominent due to its pivotal roles in the modulation of pericellular proteolysis and cancer cell proliferation [5–11]. The substrates of the MMP include an impressive range of basement membrane and extracellular matrix (ECM) macromolecules such as collagens type I, II, and III, laminins, fibronectin, fibrin and the integral membrane proteoglycan NG2 [5, 12]. The involvement of MT1-MMP in tumourigenesis in fact goes way beyond ECM degradation as the MMP is also a versatile sheddase that modulates the maturation of the regulatory proteins RANKL, MUC1 and PTK1 that are involved in cancer migration and dissemination [13–15]. Indeed, there has been well-established evidence from clinical studies over the years linking elevated MT1-MMP expression with poor prognosis in various types of cancers [16–18]. Structure-wise, MT1-MMP is a modular protein consisting of a pro-domain, a catalytic domain, a haemopexin (PEX)-like domain followed by a transmembrane (TM) anchor and a short cytoplasmic tail. Through the formation of homophilic dimers on the cell surface via its haemopexin and transmembrane domains, MT1-MMP facilitates pro-MMP-2 activation and directly promotes tumour cell invasion and proliferation [19, 20].

The enzymatic activities of the MMPs, including that of MT1-MMP, are regulated by the endogenous inhibitors, Tissue Inhibitor of Metalloproteinases (TIMPs). There are four human TIMPs (TIMP-1 to -4) and they are all small molecules ranging between 20 to 24 kDa in molecular masses [21, 22]. TIMPs exert their inhibitory functions by inserting the wedge-like “MMP-binding ridge” edges into the catalytic clefts of the MMPs in 1:1 stoichiometry to hinder the proteases from gaining access to the substrates [23–25]. Despite sharing a common three-dimensional (3D) structure, the inhibitory activity of the TIMPs is highly selective. MT1-MMP, for instance, can be inhibited by TIMP-2, -3 and -4 at sub-nanoMolar affinity but not TIMP-1 [26]. Among the TIMPs, TIMP-2 is distinct and clinically important not only because of its role as the physiological inhibitor of MT1-MMP but also for its part in pro-MMP-2 and -9 activation. By first forming a bi-molecular MT1-MMP:TIMP-2 receptor with MT1-MMP, TIMP-2 has the unique ability to interact with pro-MMP-2 (or pro-MMP-9) through its C-terminal domain in an orientation that would allow the pro-MMP-2 (or -9) for further cleavage by an adjacent, TIMP-2-free MT1-MMP [27, 28]. The release of a cascade of active gelatinases with multiple degradation activities is unfortunately, also a vital step required for neo-vascularisation and tumourigenesis [29, 30]. This undesirable feature of TIMP-2 has always been a deterring factor in its development as a therapeutic agent to treat MT1-MMP-related diseases such as cancers.

In this study, we describe a novel and highly effective fusion strategy for the exclusive purpose of MT1-MMP inhibition. By exploiting the unique predisposition of the PEX domain to dimerise on the cell surface [19, 31], we devise a warhead-and-missile tactic to target TIMP-2 to MT1-MMP using a range of soluble and membrane-anchored PEX-derived C-terminal carriers. We can reveal that a TIMP-2 variant named “T2^PEX^” created by fusion with the truncated PEX domain of MT1-MMP is highly selective as well as effective in the suppression of cellular MT1-MMP activity. Transduction of T2^PEX^ into cervical carcinoma cells not only results in a significant reduction in the invasiveness of the cells in *in vitro* settings, but also the incidence of tumour development *in vivo*. We are optimistic that the approach presented herein can represent the basis of a new strategy in TIMP engineering as well as cancer therapy.

## RESULTS

### Membrane TIMP-2s: design and rationale

**Figure 1:**
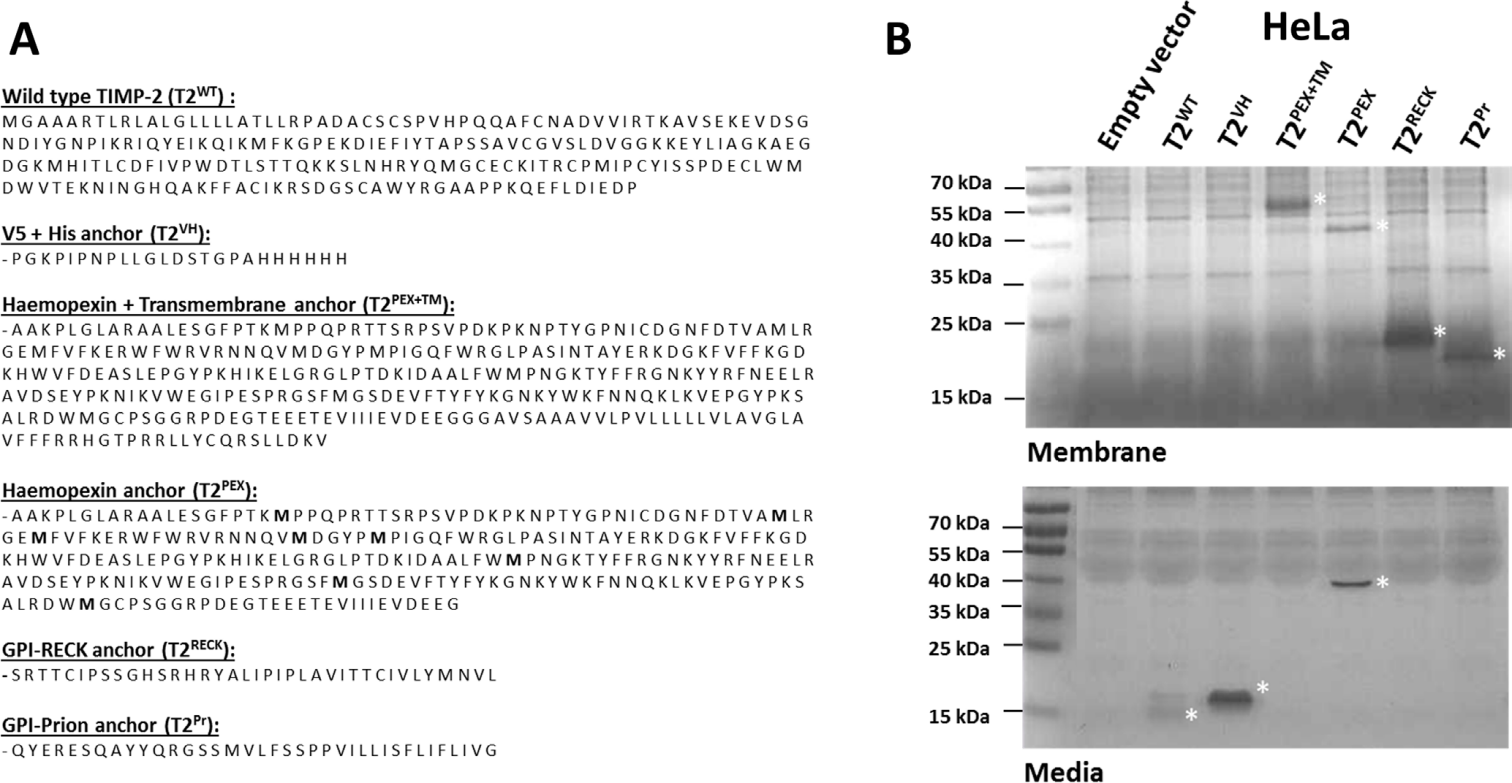
Amino acid sequences and membrane sequestration of TIMP-2s tagged with different C-terminal carriers in stably-transduced HeLa cells (**A**) Amino acid sequences for wild type TIMP-2 (T2^WT^) and the C-terminal carriers VH, PEX+TM, PEX, RECK and Pr. (**B**) Reverse zymography showing distribution of the TIMPs in the membrane fractions and conditioned media of stably-transduced HeLa cells. While T2^WT^ and T2^VH^ were entirely soluble, T2^PEX^ could be detected in both the conditioned media as well as the membrane extracts. T2^PEX+TM^, T2^RECK^ and T2^Pr^, on the contrary, were found exclusively sequestered to the membrane fractions (the TIMPs are marked by asterisks *).

A total of six different TIMP-2 chimeras were created in this study out of which three were membrane-anchored. The TIMPs are: (a) wild type TIMP-2 (T2^WT^) (b) TIMP-2 with a C-terminal V5 and 6x Histidine tag (T2^VH^) (c) TIMP-2 in fusion with the PEX and TM domains of MT1-MMP (T2^PEX+TM^) (d) TIMP-2 fused with the truncated PEX domain of MT1-MMP (T2^PEX^) (e) TIMP-2 fused to the glycosylphosphatidylinositol (GPI) signal peptide of the Reversion Inducing Cysteine Rich Protein With Kazal Motifs, i.e. RECK (T2^RECK^) and lastly, TIMP-2 fused to the GPI signal peptide of the prion protein (T2^Pr^). The amino acid sequences of the C-terminal carriers are listed in Figure 1A.

Native MT1-MMP forms dimers on the cell membrane via homophilic attraction of its PEX and TM domains [20]. Transplantation of the two domains onto TIMP-2, we hypothesised, would create a chimera TIMP (namely T2^PEX+TM^) that has the potential capacity to ensnare MT1-MMP. The soluble TIMP variant T2^PEX^ was designed primarily to investigate if the PEX domain alone was sufficient to deliver TIMP-2 to MT1-MMP in the absence of a TM anchor. T2^RECK^ and T2^Pr^ were in turn created with the sole aim of relocating TIMP-2 to the cell membrane via GPI anchors. Through anchorage to the membrane and thus being in close proximity with MT1-MMP, it was our hope that the potency of TIMP-2 against MT1-MMP could be enhanced as a consequence.

### Translocation of TIMP-2 to cell membrane by C-terminal carriers

Reverse zymography was adopted to monitor TIMP localisation at the outset of this project as the technique allows the molecular weights as well as the MMP-inhibitory activity of the chimera TIMPs to be determined [32]. Figure 1B is a reverse zymography of the membrane extracts and conditioned media of human cervical cancer cells (HeLa) stably transduced with the TIMPs. As shown, while T2^WT^ and T2^VH^ were entirely soluble, T2^PEX+TM^, T2^RECK^ and T2^Pr^ were sequestered exclusively to the membrane fraction, there was no trace of these TIMPs in the conditioned media. T2^PEX^, on the other hand, was detected in both the membrane extracts and conditioned media. The presence of T2^PEX^ in the membrane extract is a finding of particular interest to us as the TIMP should, in theory, be capable of forming complexes with MT1-MMP via its C-terminal PEX tag despite being a soluble protein. The fact that the TIMP was detectable in the membrane fraction was the first indication of its ability to bind to the target MT1-MMP as hoped.

The findings confirmed that not only had the TIMPs been expressed and folded correctly in the cells, so was their localisation. Under non-denaturing condition, the molecular masses of the TIMPs were estimated to be 16 kDa (T2^WT^), 19 kDa (T2^VH^), 45 kDa (T2^PEX+TM^), 42 kDa (T2^PEX^), 19 kDa (T2^RECK^) and 17 kDa (T2^Pr^) respectively. The estimated molecular weights were slightly lower than the calculated molecular masses of 21–55 kDa owing to the compactness of the TIMP molecule as a result of having six intramolecular disulphide bonds [24]. (More data on AAV293 and HT1080 in Supplementary Figure 1).

### Co-localisation of T2^PEX+TM^, T2^PEX^ and T2^RECK^ with MT1-MMP on the cell surface as revealed by non-permeabilised immunostaining

**Figure 2:**
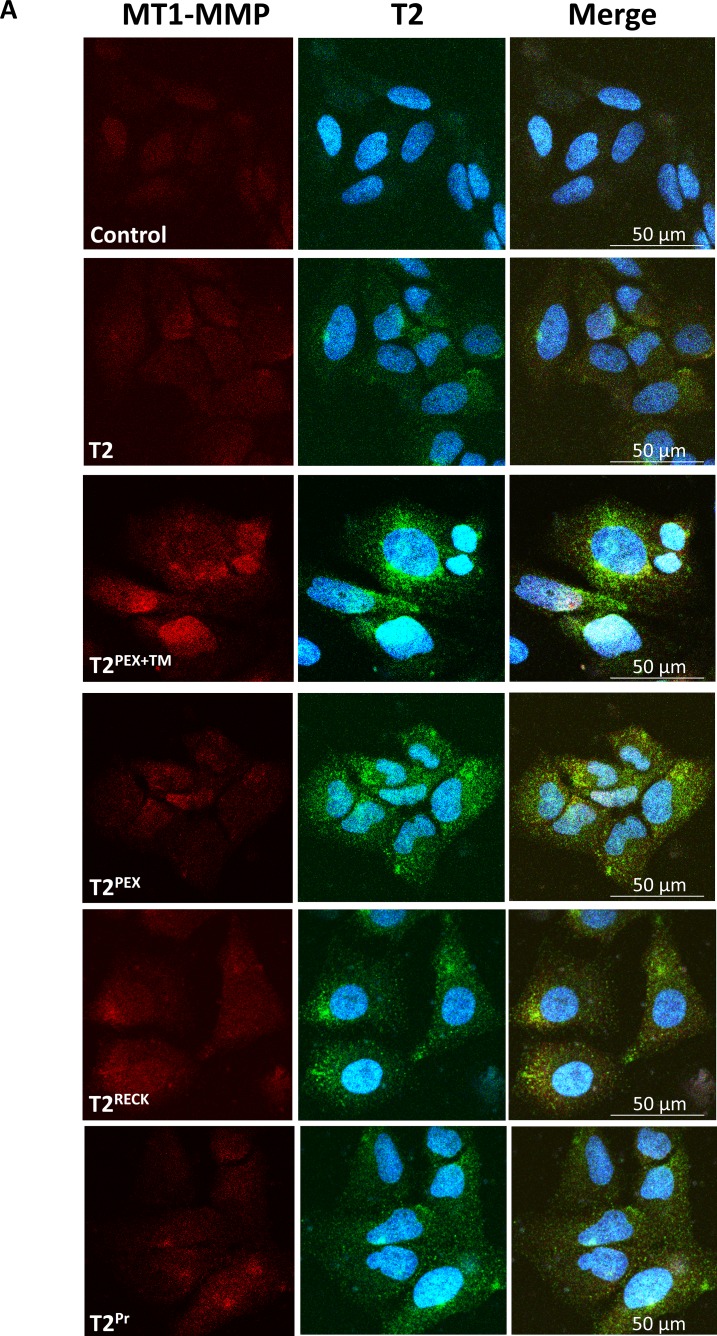
Co-localisation of membrane-anchored TIMP-2s with MT1-MMP on the cell surface as revealed by non-permeabilised immunostaining HeLa cells stably expressing T2^WT^ and T2^PEX+TM^, T2^PEX^, T2^RECK^ and T2^Pr^ were subjected to co-immunostaining with antibodies against MT1-MMP and TIMP-2 under non-permeabilising condition. While the signal of wild type TIMP-2 (T2^WT^) was faint and difficult to visualise, T2^PEX+TM^, T2^PEX^ and T2^RECK^ showed up as more intense punctate granules in clear co-localisation with MT1-MMP. In comparison to the other membrane TIMP-2s, T2^Pr^ was lower in expression and thus harder to distinguish from that of T2^WT^ (Left: MT1-MMP antibody; Middle: TIMP-2 antibody in conjunction with 4′, 6′-diamidino-2-phenylindole (DAPI) staining; Right: merged image).

We next performed a double immunostaining on stably-transduced HeLa cells under non-permeabilising condition to ascertain if the TIMPs had indeed been successfully translocated to the cell surface. As shown in Figure 2, not only could T2^PEX+TM^, T2^PEX^ and T2^RECK^ be detected as patchy and small punctate granules on the cell membrane, the TIMPs also demonstrated clear signs of co-localisation with MT1-MMP. In HeLa cells expressing T2^PEX+TM^, the signal of MT1-MMP appeared to be slightly higher possibly as a result of MT1-MMP:T2^PEX+TM^ complex accumulation on the cell surface. Comparing to the other TIMPs, T2^Pr^ was also detectable albeit at a lower intensity. Moreover, the TIMP appeared to show a less obvious sign of co-localisation with MT1-MMP probably because of the lower expression level.

### T2^PEX+TM^ and T2^PEX^ induce “mesenchymal to amoeboid-like” morphological transition in HT1080 cells

**Figure 3:**
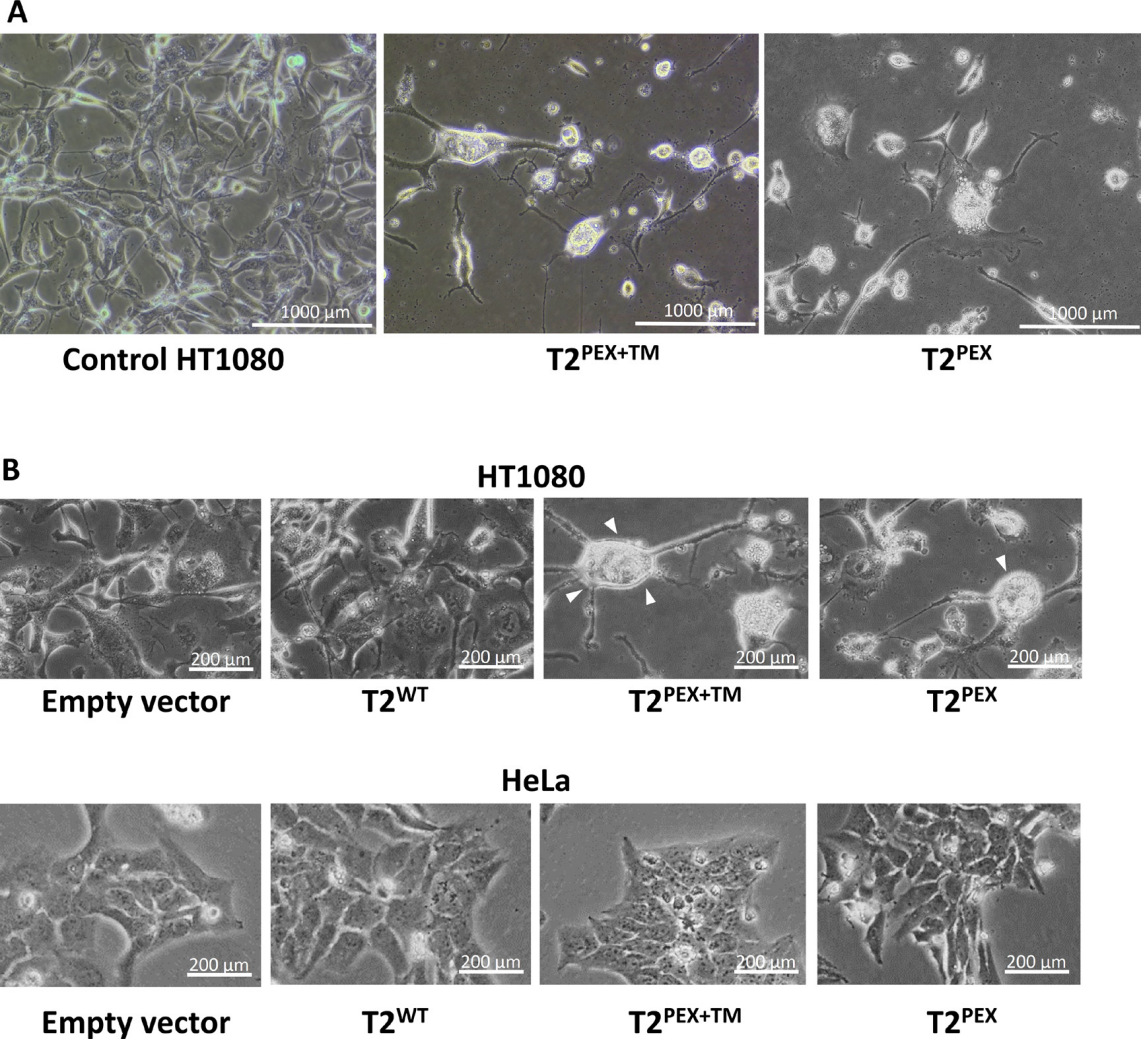
T^2PEX+TM^- and T2^PEX^-induce mesenchymal to amoeboid-like morphological transition in HT1080 cells (**A**) Transduction of T2^PEX+TM^ and to a lesser extent, T2^PEX^ into HT1080 induced a morphological change from spindle-shaped to a more rounded amoeboid-like phenotype (**B**) Top panel: Detailed image of the amoeboid-like cell phenotype induced by T2^PEX+TM^ and T2^PEX^ expression (highlighted by arrowheads). Lower panel: no shape change was observed in HeLa cells similarly transduced with the TIMPs.

An interesting feature we noticed in HT1080 cells stably transduced with T2^PEX+TM^ and to a smaller degree, T2^PEX^, was their heterogeneous size but essentially more rounded and amoeba-like appearance (Figure 3A). As highlighted in Figure 3B, the cell shape of HT1080 expressing T2^PEX+TM^ and T2^PEX^ was distinctly different from the spindle-shaped control cells (Figure 3B, top panel). The change in phenotype observed in T2^PEX+TM^ and T2^PEX^ cells was rather similar to the mesenchymal-to-amoeboid-like morphological transition previously documented in proteolytically-disabled HT1080 cells [33]. By constraining the proteolytic ability of HT1080 cells with a cocktail of protease inhibitors, Wolf and co-workers first noticed that HT1080 cells were able to switch from a “proteolytically-active mesenchymal type movement” to a “non-proteolytic amoeboid movement” as a means of escaping abrogation of pericellular proteolysis. The fact that T2^PEX+TM^- and T2^PEX^-expressing HT1080 cells also assumed an amoeboid-like morphology is a putative reflection of the proteolytically disabled state of the cells. Interestingly, no cell shape change was noticed in HeLa cells similarly transduced with the two chimera TIMPs (Figure 3B, lower panel).

### Co-localisation of T2^PEX+TM^ and T2^PEX^ with intracellular MT1-MMP

**Figure 4:**
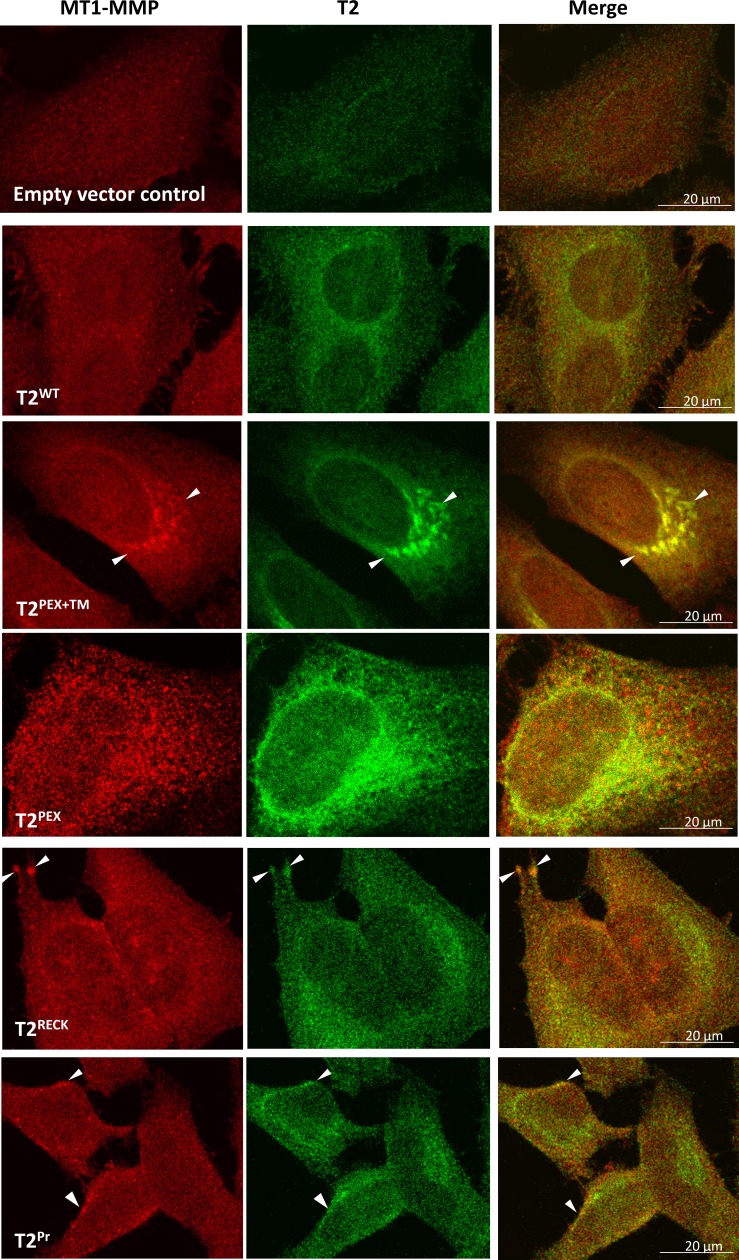
Co-localisation of T2^PEX+TM^ and T2^PEX^ with intracellular MT1-MMP in HeLa cells as revealed by confocal microscopy While T2^WT^, T2^RECK^ and T2^Pr^ only partially co-localised with MT1-MMP, the co-localisation of T2^PEX+TM^ and T2^PEX^ was intense. T2^PEX+TM^ in particular formed aggregates with MT1-MMP in areas around the nucleus (highlighted by arrowheads).

To visualise how the membrane-anchored TIMP-2s were localised inside the cells, a double immunostaining was subsequently carried out on HeLa cells as described above except under permeabilising condition. As shown in Figure 4, T2^PEX+TM^ and T2^PEX^ exhibited an intense degree of co-localisation with cellular MT1-MMP in the cytoplasm and perinuclear regions. In T2^PEX+TM^-expressing cells, masses of T2^PEX+TM^:MT1-MMP aggregates could be detected in areas around the nucleus (Figure 4, highlighted by arrowheads). The co-localisation profile of T2^PEX^ was slightly different. Instead of forming granule-like aggregates, T2^PEX^:MT1-MMP complexes were generally smaller in size but flaky in appearance. In contrast, the co-localisation pattern of the other two membrane TIMP-2s namely T2^RECK^ and T2^Pr^ was much less pronounced. The duo co-localised with MT1-MMP primarily in the cytoplasm but also on the cell membrane to a lesser extent (highlighted by arrowheads). Overall, the degree of brightness observed in T2^RECK^ and T2^Pr^ was far lower than those of T2^PEX+TM^ and T2^PEX^. In comparison with the membrane-anchored TIMP-2s, wild type TIMP-2 (T2^WT^) showed only a limited co-localisation with MT1-MMP mainly at the perinuclear regions.

### T2^PEX+TM^ interacts with intracellular MT1-MMP to end up in the aggresomes

As opposed to T2^PEX+TM^, the co-localisation pattern of T2^PEX^ was limited to the “flaky” phase. At no time could we detect granule-like aggregates in the cells.

### HT1080 and HeLa cells display different gelatinolysis patterns

Prior to assessing the inhibitory potency of the individual chimera TIMP-2s, we first investigated the MT1-MMP activity of our cell lines of choice by monitoring their profiles of gelatin degradation on a thin layer of fluorescent gelatin substratum. As highlighted by Figure 6A, the film on which HT1080 cells were cultured was typically marred by distinctive trails of dark imprints that in a way, reflect the subcellular location in which MT1-MMP was most enzymatically active. HeLa cells displayed an entirely different pattern of gelatin degradation. Instead of leaving imprints on the substratum, the cells were more characteristically surrounded by shady halos of gelatin-free zones as highlighted by arrowheads the same figure. We further immunostained HT1080 cells with an antibody against MT1-MMP and matched the subcellular distribution of the proteinase to the gelatinolytic imprints left on a fluorescent gelatin film. The overlay image (Figure 6B, 6C) indicates that although MT1-MMP was present most abundantly around and under the nucleus as well as the lamellipodia, the gelatinolytic activity was overwhelmingly concentrated around and beneath the perinuclear region.

**Figure 5:**
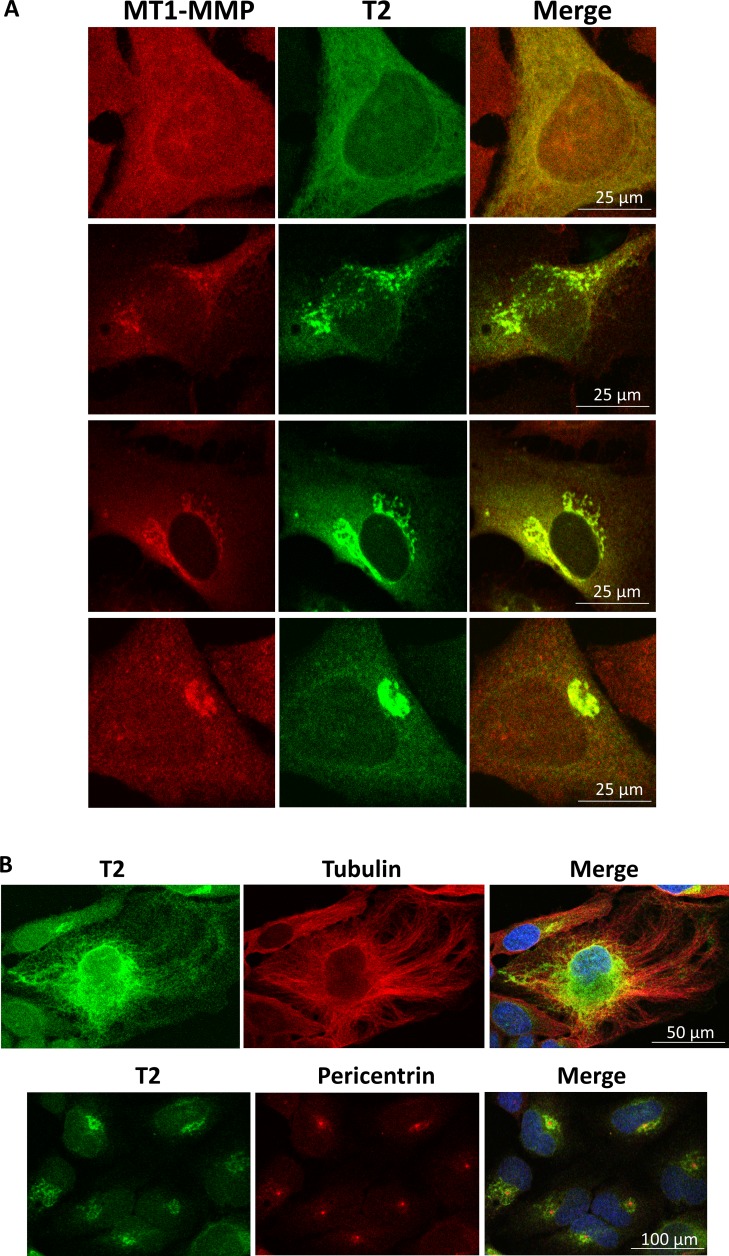
Confocal microscope images showing different form of T2^PEX+TM^:MT1-MMP aggregates in HeLa cells (**A**) From nebulous flakes in the cytoplasm (top panel) to granule-like aggregates beside the nucleus (lowest panel), different forms of T2^PEX+TM^:MT1-MMP aggregates could be detected in HeLa cells stably transduced with T2^PEX+TM^. (**B**) Top panel: Double immunostaining with antibodies against TIMP-2 and tubulin reveals the journey of T2^PEX+TM^ micro-particulates to the centrosome via the microtubule transportation network. Lower panel: assembly of T2^PEX+TM^ aggresome at the centrosome as shown by immunostaining with antibodies against TIMP-2 and pericentrin, a centrosome marker. The cell nuclei were highlighted by DAPI staining

**Figure 6:**
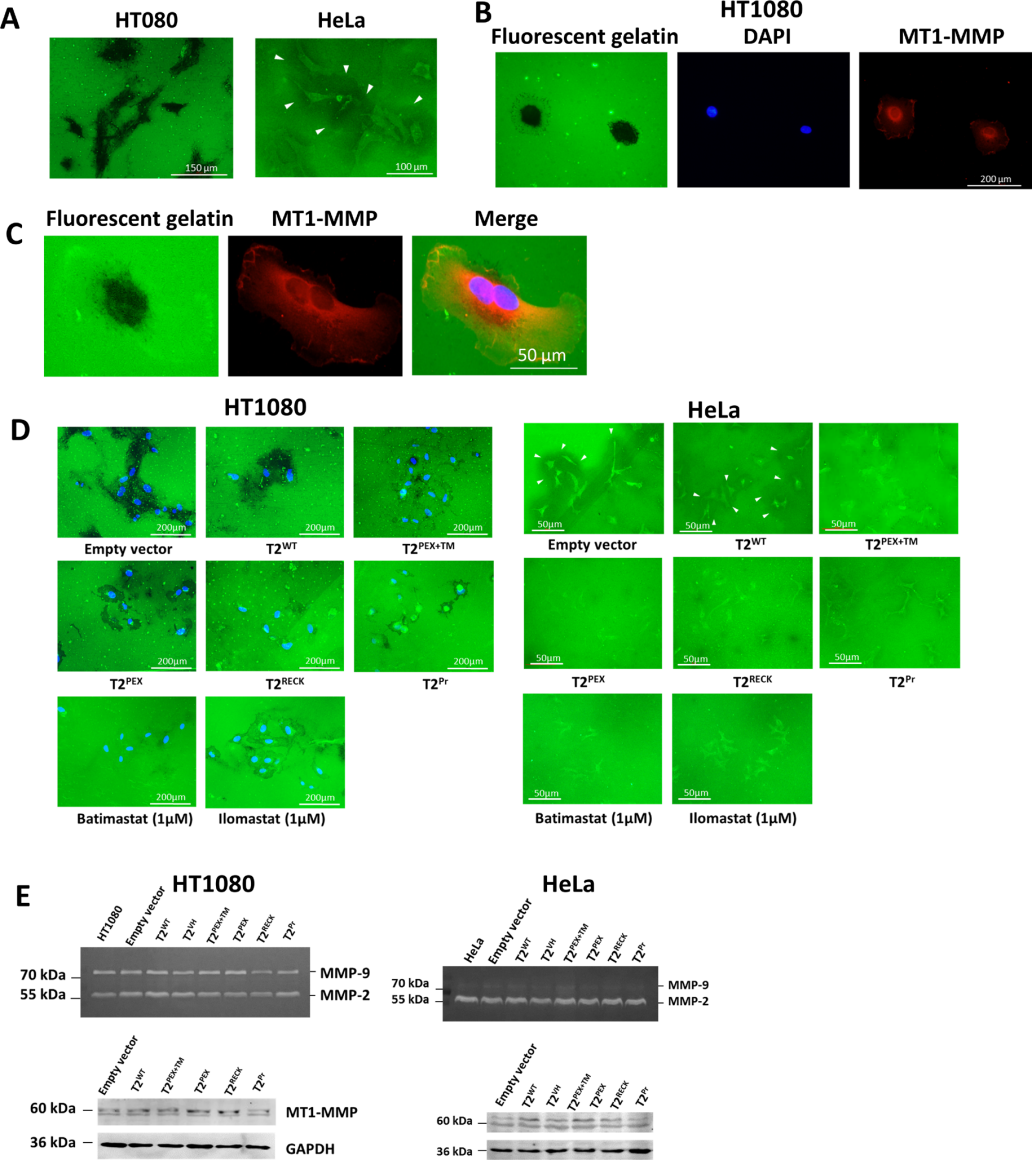
Membrane-anchored TIMP-2s inhibit gelatin degradation by HT1080 and HeLa cells (**A**) While HT1080 cells typically leave a criss-cross trail of imprints along their tracks of movement, the gelatinolysis activity of HeLa cells appears as clear pericellular halos against a green background (marked by arrowheads). (**B**) Left: imprints left by HT1080 cells on fluorescent gelatin film after an overnight incubation. Right: subcellular localisation of MT1-MMP as revealed by immunostaining. Middle: DAPI staining of the nuclei. (**C**) Magnified images demonstrating how the gelatinolytic activity of HT1080 (left) correlates with the subcellular distribution of MT1-MMP (middle). Superimposition of the images shows that the gelatinolysis activity of HT1080 was largely concentrated around and beneath the nucleus (right). (**D**) In both HT1080 and HeLa cells, membrane TIMP-2-expressing cells had significantly smaller imprints/halos in comparison to the control and T2^WT^-expressing cells. (**E**) no significant changes in MMP-2, -9 and MT1-MMP levels were detected among membrane TIMP-2-expressing cells as shown by zymography and western blot.

### Membrane TIMP-2s impair the gelatinolytic activity of HT1080 and HeLa cells

We next assessed the inhibitory activity of the membrane-anchored TIMP-2s by comparing the gelatinolysis pattern of stably-transduced HT1080 and HeLa cells using the protocol described above. As demonstrated in Figure 6D, HT1080 cells transduced with T2^WT^ left a trail of imprints similar in size and intensity to those of the empty vector control. In contrast, the gelatinolytic activity of all the membrane-anchored TIMP-2-producing cells were significantly weaker in particular T2^RECK^. Though not as obvious, T2^PEX+TM^, T2^PEX^ and T2^Pr^-expressing cells also displayed a significant reduction in activity in comparison with T2^WT^ and the control. The broad-range MMP inhibitors batimastat (1 μM) and ilomastat (1 μM) were included in the experiment to confirm that the gelatin-degradation activity was indeed MMP-related.

On the whole, the profile of inhibition observed in HeLa is comparable to that of the HT1080. While the control and T2^WT^-expressing HeLa cells were surrounded by a zone of shady halos against a green background, no such halos existed around the cells that have been transduced with T2^PEX^ and T2^RECK^. On the other hand, although smaller and less intense halos were occasionally spotted around T2^PEX+TM^ and T2^Pr^ cells, the contrast with the background was so weak that they could barely be noticed in the majority of the cell population. In all, results from the assay corroborated our previous findings on the potency of the chimera TIMP-2s on MT1-MMP inhibition.

### Membrane-anchored TIMP-2s have no effect on pro-MMP-2, -9 activation and MT1-MMP expression

The ensuing question we wanted to address is whether membrane-anchored form of TIMP-2s have an effect on pro-MMP-2 and -9 activation. Figure 6E contains images of zymography gels on which the conditioned media of stably-transduced HT1080 and HeLa cells were analysed. In HT1080 as well as HeLa, an almost indistinguishable pattern of MMP-2 and -9 processing was observed among the controls and all the TIMP-expressing cells.

To examine if the membrane-anchored TIMP-2s had impacted on MT1-MMP expression, the cell lysates were subjected to western blot analysis with an antibody against the catalytic domain of MT1-MMP. In HT1080, only two major forms of MT1-MMP were detected, i.e. a 60 kDa variant and a smaller species of approximately 58 kDa (Figure 6E, left). Apart from a slight reduction in the 60 kDa variant in T2^Pr^, there was no indication to suggest that MT1-MMP production has been considerably altered among the cells. In HeLa, the two MT1-MMP variants detected were of 60 kDa and 63 kDa in mass (Figure 6E, right). Again, no substantial difference in MT1-MMP expression was noticed among the samples. The results thus indicated to us that membrane TIMP-2s had no significant impact on either pro-MMP-2/-9 activation or MT1-MMP production.

### Impaired migration of T2^PEX+TM−^, T2^PEX−^ and ^T2RECK^-expressing HT1080 and HeLa cells in Transwell^®^ migration assay

**Figure 7:**
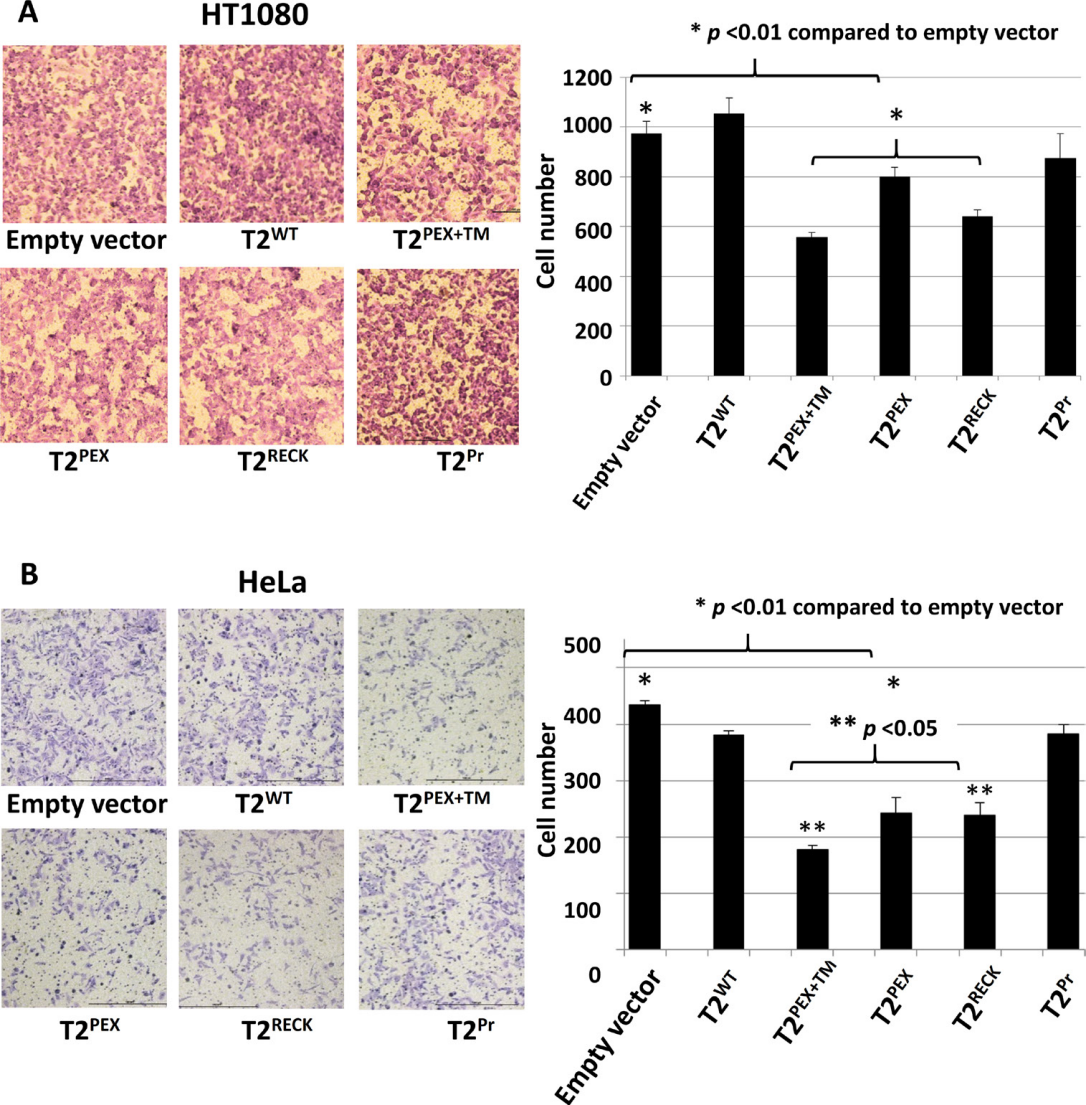
T2^PEX+TM^, T2^PEX^ and T2^RECK^ suppress HT1080 and HeLa cell migration in Transwell^®^ invasion assays In both cell types, T2^PEX+TM^-expressing cells demonstrated the slowest migration rate closely followed by T2^RECK^ and T2^PEX^ (*p < 0.01 compared to empty vector control. The readings reflect the average of two technical repeats). Y-axis: number of cells that had traversed the membrane after an overnight incubation.

A Transwell^®^ migration assay was set up to evaluate if the migratory behaviour of HT1080 and HeLa cells had been affected by the membrane-anchored TIMP-2s. The results of the assay are summarised in Figure 7. In both cell lines, T2^PEX+TM^ emerged as the most effective TIMP in the suppression of cell migration (*p < 0.01 compared to empty vector control and T2^WT^) closely followed by T2^RECK^ and T2^PEX^. T2^Pr^ fared worst in this assay, the TIMP had a negligible impact on cell motility in both HT1080 and HeLa cells. Of noteworthy is the near-identical profile of inhibition displayed by the two cell lines.

### Suppression of cervical cancer (HeLa) growth by T2^PEX+TM^ and T2^PEX^ *in vivo*

**Figure 8:**
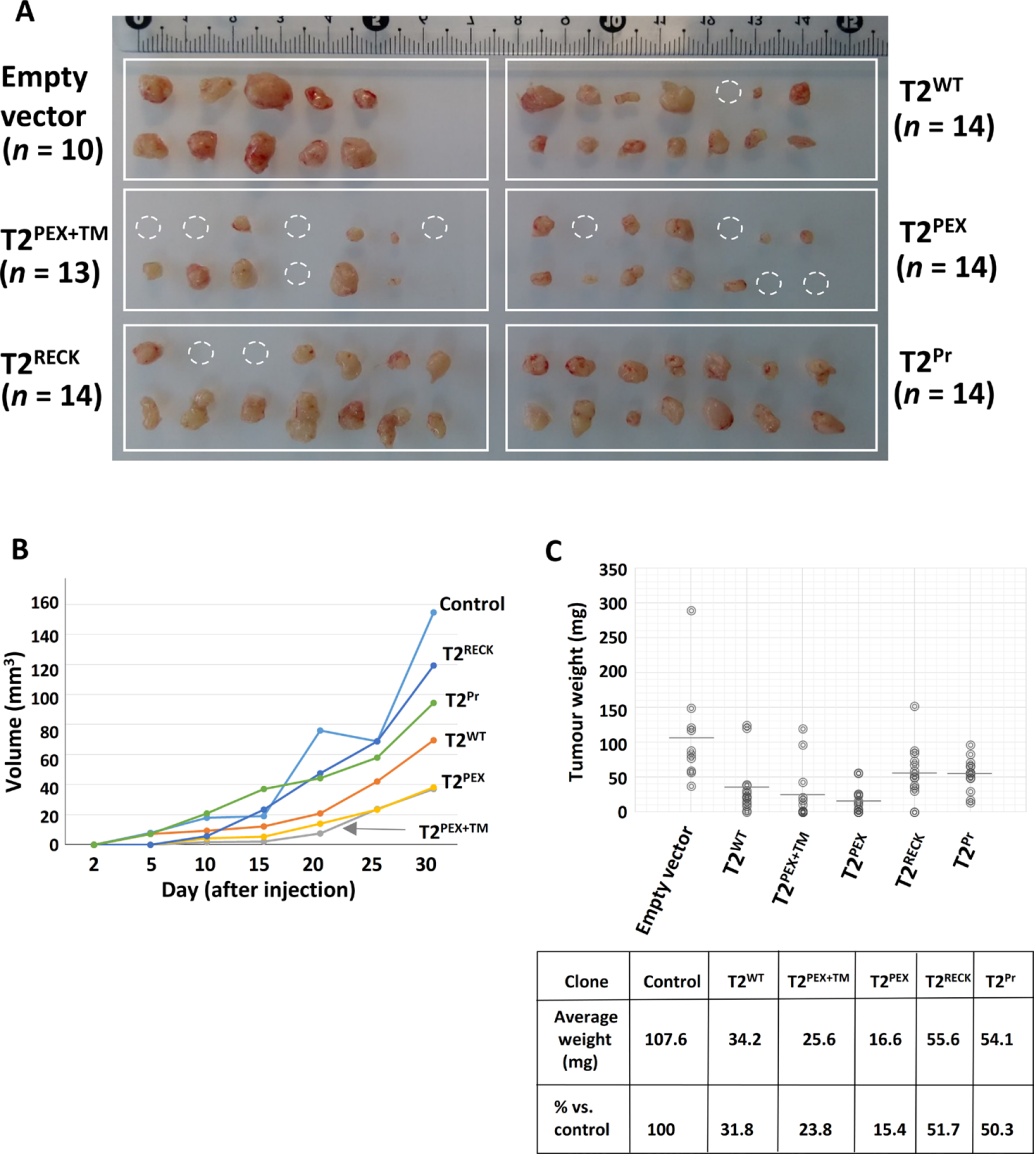
T2^PEX+TM^ and T2^PEX^ suppress cervical carcinoma cell growth *in vivo* (**A**) While all the inoculums in the empty vector control and T2^Pr^ groups developed into tumours (100%), much lower tumour development incidence were recorded in T2^PEX+TM^ (8 out of 13; 61.5%) and T2^PEX^ (10 out of 14; 71.4%) mice. Dotted circles indicate inoculums that failed to become tumours. (**B**) Tumour grow curves measured at different time points over a 30-day period. Plotted data represent the mean for each TIMP group. (**C**) Scattered chart showing individual tumour masses on day-30 when the experiment was concluded (grey bars indicate the mean for each group). On average, the tumour masses for T2^PEX+TM^ (25.6 mg) and T2^PEX^ (16.6 mg) mice were significantly smaller than the control (107.6 mg) group (*p < 0.001). The study has been performed twice in different scale to confirm the reproducibility of the results.

The inhibitory activities of the TIMPs were further evaluated *in vivo* by subcutaneous implantation of all the TIMP-producing HeLa cells into NOD/SCID mice. Figure 8A summarises the results of the study on day-30 when the experiment was concluded. As shown, all the 10 inoculums in the control (empty vector) group grew to an average volume of 155 mm^3^ over the course of 30 days. In comparison, tumours from the TIMP groups were substantially smaller. The growth curve contained in Figure 8B indicates that, while primary tumours started to emerge within 10 days of implantation in the control and T2^Pr^ groups, there was barely any evidence of tumour formation in the remaining mice. Rapid growth occurred between day-10 and 20 in the control, T2^Pr^ as well as T2^RECK^ groups with tumours in these mice showing an accelerated expansion in volume. Conversely, only small tumours appeared in the T2^WT^, T2^PEX+TM^ and T2^PEX^ mice by the end of day-20. Tumour volumes continued to increase rapidly in the control, T2^Pr^ and T2^RECK^ mice between day-20 to 30 when the experiment reached a humane endpoint. In the same time, mice in the T2^WT^ group also recorded a surge in growth rate. T2^PEX+TM^ and T2^PEX^ tumours, in comparison, were still developing at a relatively modest pace by the time the experiment was terminated. In fact, the estimated average tumour volumes of the two (37 mm^3^) was only 24% of that of the control (155 mm^3^) (*p < 0.001).

Post-mortem analysis carried out on day-30 confirmed that, without exception, the average tumour masses of the TIMP groups were all significantly smaller than that of the control group (*p < 0.05). Figure 8C is a scattered chart showing the individual masses for all the tumours in this study. Among the TIMPs, the performance of T2^PEX+TM^ and T2^PEX^ were the most impressive. Not only did the pair have the lowest tumour development incidence; only 8 of the 13 (61.5%) implantations in the T2^PEX+TM^ group and 10 of the 14 (71.4%) in the T2^PEX^ group developed into tumours by the end of this study, the average tumour masses of the two (25.6 mg for T2^PEX +TM^; 16.6 mg for T2^PEX^) were only a fraction of those of the control mice (mean tumour mass = 107.6 mg) (*p < 0.001). Comparing to T2^WT^, T2^PEX^ was also a more effective tumour suppressor (*p < 0.1).

On the other hand, even though T2^RECK^ and T2^Pr^ fared considerably better than the control group in this study, their performance was less than satisfactory in comparison with that of T2^WT^. With average tumour masses of 55.6 mg and 54.1 mg, the duo were indeed poorer growth inhibitors than T2^WT^ (mean tumour mass = 34.2 mg) (*p < 0.1).

## DISCUSSION

Given its pivotal role in tumour dissemination, MT1-MMP has been the subject of a variety of intervention strategies over the years. Therapeutic approaches ranging from small molecule inhibitors (MMPI), blocking antibodies to recombinant TIMPs, to name a few, are among the tactics that have been extensively explored and pursued [34–38]. While the MMPI have suffered a setback in advanced clinical trials due to unforeseen toxicity-related side effects, progress with the TIMPs has been slow [39]. The main reason, from a structural point of view, comes from the catalytic domains of the MMP. Owing to a general resemblance in the 3D structure of the catalytic domains of the Metzincins, it has so far not been possible to design a TIMP to be exclusively MT1-MMP-specific. The protein engineering work in this study differs from previous mutagenesis endeavours as our current approach aims to improve the efficiency of TIMP-2 not by mutating the TIMP as customarily performed but by redesigning its mode of delivery.

Collectively, evidence from reverse zymography on membrane extracts and non-permeabilised immunostaining confirm that the three carriers PEX+TM, RECK and Prion have all fulfilled their missions of providing anchorage in the way that we had anticipated. The fourth carrier PEX is not intended to be a membrane anchor. Instead, the carrier was designed to entice and ensnare MT1-MMP to form a homophilic complex with TIMP-2. The fact that T2^PEX^ is partially partitioned to the membrane and its intense co-localisation with MT1-MMP inside the cell as well as on the cell surface as revealed by immunofluorescence corroborates our belief that the carrier has also achieved its purpose of complexing with MT1-MMP. The first sign indicating the effectiveness of the PEX carriers comes from the mesenchymal-to-amoeboid-like morphological conversion observed in HT1080 cells. Transduction of the TIMPs into HT1080 is, in effect, very similar to that of adding a cocktail of MMP inhibitors as described by Wolf and colleagues [33]. The proteolytically attenuated state of T2^PEX+TM^ and T2^PEX^-expressing cells, as evidenced by their inability to digest gelatin substratum and diminished motility across Transwell^®^ chambers, further validates the inhibitory efficacy of the TIMPs.

In all, findings from reverse zymography, cell morphology, gelatinolytic and co-localisation studies all lead to the conclusion that T2^PEX+TM^ has been processed to a membrane-anchored form recognisable by its intended target MT1-MMP. Through interactions at the TIMP, PEX and perhaps the TM domains, T2^PEX+TM^ forms a membrane-bound and yet non-functional protease:inhibitor complex with MT1-MMP that ultimately ends up in the recycling pathway. Much unexpected, however, is its exceptional ability to interact and co-aggregate with the protease inside the cells. Aggresome normally forms when the amount of intracellular misfolded proteins exceeds the clearance capacity of the chaperone-mediated refolding and proteasomal degradation systems [40]. Given that TIMP:MT1-MMP aggresomes are present only in T2^PEX+TM^-producing cells and not T2^PEX^, the uniqueness of T2^PEX+TM^ can therefore be attributable to the TM anchor. The fact that co-aggregation with MT1-MMP only occurred in T2^PEX+TM^ may also reflect the more demanding recycling processes required for misfolded membrane proteins as opposed to soluble cytoplasmic ones. The efficacy of T2^PEX+TM^ and T2^PEX^ in abrogating tumour development *in vivo*, we surmise, is due to their ability to prevent MT1-MMP-driven cleavage and remodelling of the cell matrices by the implanted cancer cells. A precondition for MT1-MMP to be a tumour-derived growth factor is pericellular proteolysis of the ECM [41]. Deprived of the capability to proteolytically change the extracellular matrices, T2^PEX+TM^- and T2^PEX^-expressing HeLa cells are therefore trapped in a compact, spherical configuration incapable of undergoing changes in cell shape or cytoskeletal reorganization required for 3D growth.

Besides an improvement in delivery, our current strategy may also offers several clear advantages over the conventional way of TIMP engineering. First and foremost, by immobilising TIMP-2 to the cell membrane, no secretion of the inhibitor to the media was permitted. Wastage through discharge to the conditioned media is therefore kept to a minimum. Secondly, our strategy may have also considerably enhanced the affinity of TIMP-2 for MT1-MMP. By its own, the association constant (*K*_i_^app^) of TIMP-2 for the catalytic domain of MT1-MMP is 1.3 nM [26]. The affinity of the PEX-tagged T2^PEX+TM^ and T2^PEX^ are likely to be even higher due to an additional interaction site in their C-terminal domains [20, 31]. Thirdly, our approach has overcome the shortcoming of TIMP-2 as a pro-MMP-2 activator, a defect that has thus far compromised its prospect as a therapeutic agent [28, 42, 43]. Due to the bulky and restrained nature of their C-terminal domains, our modelling exercise suggests that membrane-anchored forms of TIMP-2 are sterically-hindered from forming a trimolecular complex with MT1-MMP and pro-MMP-2 that could lead to subsequent pro-MMP-2 potentiation. Furthermore, embedment to the membrane might also be beneficial as a means of reducing TIMP-2's exposure to circulating proteases in the extracellular compartment.

In all, we have provided concrete evidence to demonstrate the effectiveness of fusing TIMP-2 with the PEX domain as a means of delivering the TIMP for MT1-MMP inhibition. T2^PEX+TM^ and T2^PEX^ have both achieved the goal of interacting with MT1-MMP in the cell and the process has led to attenuation of the protease both *in vitro* and *in vivo*. The findings further underscore the prospect of MT1-MMP inhibition as a viable option to block cancer proliferation. We are currently in the process of producing and testing the therapeutic efficacy of recombinant T2^PEX^ protein in mice. The results will be submitted for publication upon conclusion of the project in the near future. It is our hope that the results would form the basis of a novel and targeted approach in cancer inhibition.

## MATERIALS AND METHODS

### Materials

All reagents, restriction enzymes and protein extraction kit were products of ThermoScientific USA unless otherwise stated. Antibodies against TIMP-2 (R&D Systems; MAB971) and MT1-MMP (Abcam; Ab72685 against activated MT1-MMP from cleavage adjacent to Tyr112) were purchased separately from R&D Systems, MN and Abcam, MA, USA. *Pwo* DNA polymerase and ProteoExtract^®^ Native Membrane Protein Extraction Kit were products of Merck, Darmstadt, Germany. TIMP-1 and -2 proteins were produced in house using Sf-9 insect cell as described in our previous paper [44]. AAV293, HeLa and HT1080 cell lines were purchased from the Shanghai Cell Bank, Chinese Academy of Science while Lenti-X 293T cells were imported directly from Takara^®^ BIO Inc., CA without further authentication. Non-obese diabetic/severe combined immunodeficiency (NOD/SCID) mice were ordered through GenePharma Co. Ltd, Biobay, Suzhou Industrial Park, Jiangsu, China. All the experiments in this study have been performed independently at least twice to confirm the reproducibility of the findings.

### Construction of membrane-anchored TIMP-2s

Short carriers consisting of forty amino acid residues or fewer were introduced directly to the C-terminus of TIMP-2 by overlapping polymerase chain reaction (PCR) with reverse primers bearing the corresponding sequences. For longer carriers such as PEX +/− TM domains, the DNA fragments encoding for TIMP-2 and MT1-MMP PEX domain were first amplified as independent fragments by PCR before ligation into pcDNA3.1 vector. All the clones generated in this study had been sequenced in both strands to confirm that no unwanted mutation had been introduced into the cDNAs during PCR and cloning processes.

### Stable transfection using Lentivirus

Lentivirus used for infecting HT1080 and HeLa cells were generated with Lenti-X Packaging Single Shots^®^ system in Lenti-X 293T cells following the recommended procedures by Takara^®^ BIO Inc. Upon transduction, selection of cells was performed by adding 1 μg/mL of puromycin to the culture media until stable cell lines were obtained, usually after 2 weeks of culture.

### Membrane protein extraction

Briefly, TIMP-2-expressing cells were first dislodged from 6-well culture plates and washed twice with ice-cold 1× phosphate buffer saline (PBS) supplemented with 5 mM EDTA before extraction with 200 μL buffer I (soluble proteins) and 100 μL buffer II (membrane proteins) at 4°C as per manufacturer's instructions. Extracted membrane proteins were stored in 50 μL aliquots at −80°C until required for analysis.

### Reverse zymography

Reverse zymography was carried out in 11% non-denaturing SDS-PAGE incorporated with 0.3% gelatin and MMP-2 essentially as described [32]. Following electrophoresis, gels were washed three times in 2.5% Triton X-100 solution followed by a further three washes in 2.5% Triton X-100 in 50 mM Tris HCl, pH 7.5. After a brief rinse in running water, the gels were incubated overnight at 37°C in an activation buffer (50 mM Tris HCl pH7.5, 50 mM NaCl, 10 mM CaCl_2_ and 10 μM ZnCl_2_) before staining with 0.3% Coomassie Blue in 20% v/v methanol, 1% acetic acid in distilled water.

### Transwell^®^ migration assay

For Transwell^®^ assay, 5 × 10^4^ cells were seeded in duplicate on the upper layer of Transwell^®^ Permeable Support inserts (8 μm pores) in serum-free DMEM while the outer compartments (12-well plate) were filled with DMEM supplemented with 5% fetal calf serum (FCS). Following overnight incubation at 37°C, the cells that had traversed the insert was fixed, permeabilised with 4% formaldehyde/methanol and stained with Giemsa dye for 15 minutes. The number of migrated cells were counted from three different fields per-Transwell^®^ and the results are expressed as the average of two technical repeats. The experiment had also been repeated twice at different cell density (10 and 15 × 10^4^ cells/Transwell^®^) to ensure the reproducibility of the profile of migration.

### Immunofluorescence

For immunofluorescence under permeabilising condition, cells cultured in Nunc^®^ chamber slides were first treated with 4% formaldehyde in PBS for 5 minutes followed by blocking for 2 hours in PBS containing 5% BSA and 0.3% TX-100 before incubation in primary antibodies (1:100 in dilution buffer containing 1% BSA, 0.3% TX-100 in PBS) at 4°C overnight. Following a 15-minute wash with PBS, Alexa Fluor^®^ 488 (or 555) anti-mouse and/or anti-rabbit fluorophore-conjugated secondary antibodies (1:1,000 in the same dilution buffer) were added to the cells for 2 hours before the chamber slides were washed and mounted in ProLong^®^ Antifade reagent and examined under a Nikon Eclipse Ti confocal microscope. The same experimental procedure was used for non-permeabilising immunofluorescence except no TX-100 was added to the blocking and antibody dilution buffers.

### Gelatin degradation assay

Approximately 500 cells were seeded in Nunc^®^ Lab-Tek II Chamber Slides^®^ pre-coated with porcine Oregon Green^®^ 488-conjugated gelatin (0.5 mg/mL). Following overnight incubation at 37°C, the cells were fixed in 4% paraformaldehyde and mounted in ProLong^®^ Antifade reagent before examination with a Nikon Eclipse Ni inverted fluorescent microscope.

### Tumour development study in NOD/SCID mice

All animal experiments were conducted in GenePharma Co. Ltd, Biobay, Suzhou Industrial Park, China (licenced by Jiangsu Provincial Government: Licence number SYXK (Su) 2014–0054) in strict accordance to the protocols outlined in the National Guidance for Animal Care, China with respect to husbandry, veterinary care and experimental procedures throughout the entire length of the project. Immediately before injection, HeLa cells were trypsinised, washed twice with PBS and re-suspended to yield 1 × 10^6^ cells/mL in cold DMEM without FCS. Suspended cells (5 × 10^5^) were delivered by subcutaneous injection into the left and right flanks of 6-week-old female NOD/SCID mice, where *n* varied between 10 and 14 per group. Mice were monitored for tumour formation over 30 days with digital callipers. Tumour volume was determined with the formula: *Volume* = *Length* x (*Width)*^2^ x π/6, where the width is the smaller of the two perpendicular diameters.

## SUPPLEMENTARY MATERIALS FIGURES AND TABLES



## Abbreviations

GPI: glycosylphosphatidylinositol
MMP: Matrix metalloproteinase
MT1-MMP: Membrane type-1 MMP
PEX: haemopexin
TIMP: Tissue inhibitor of metalloproteinase
TM: transmembrane
