# Supplementary material for: Ensnaring membrane type 1-matrix metalloproteinase (MT1-MMP) with tissue inhibitor of metalloproteinase (TIMP)-2 using the haemopexin domain of the protease as a carrier: a targeted approach in cancer inhibition
Source: Oncotarget. 2017 Feb 7;8(14):22685–99. doi: 10.18632/oncotarget.15165 (PMC5410255; doi:10.18632/oncotarget.15165)
Supplement: Supplementary file 1 [file oncotarget-08-22685-s001.pdf]

Ensnaring membrane type 1-matrix metalloproteinase (MT1-MMP) with tissue inhibitor of metalloproteinase (TIMP)-2 using the haemopexin domain of the protease as a carrier: a targeted approach in cancer inhibition

Supplementary Materials

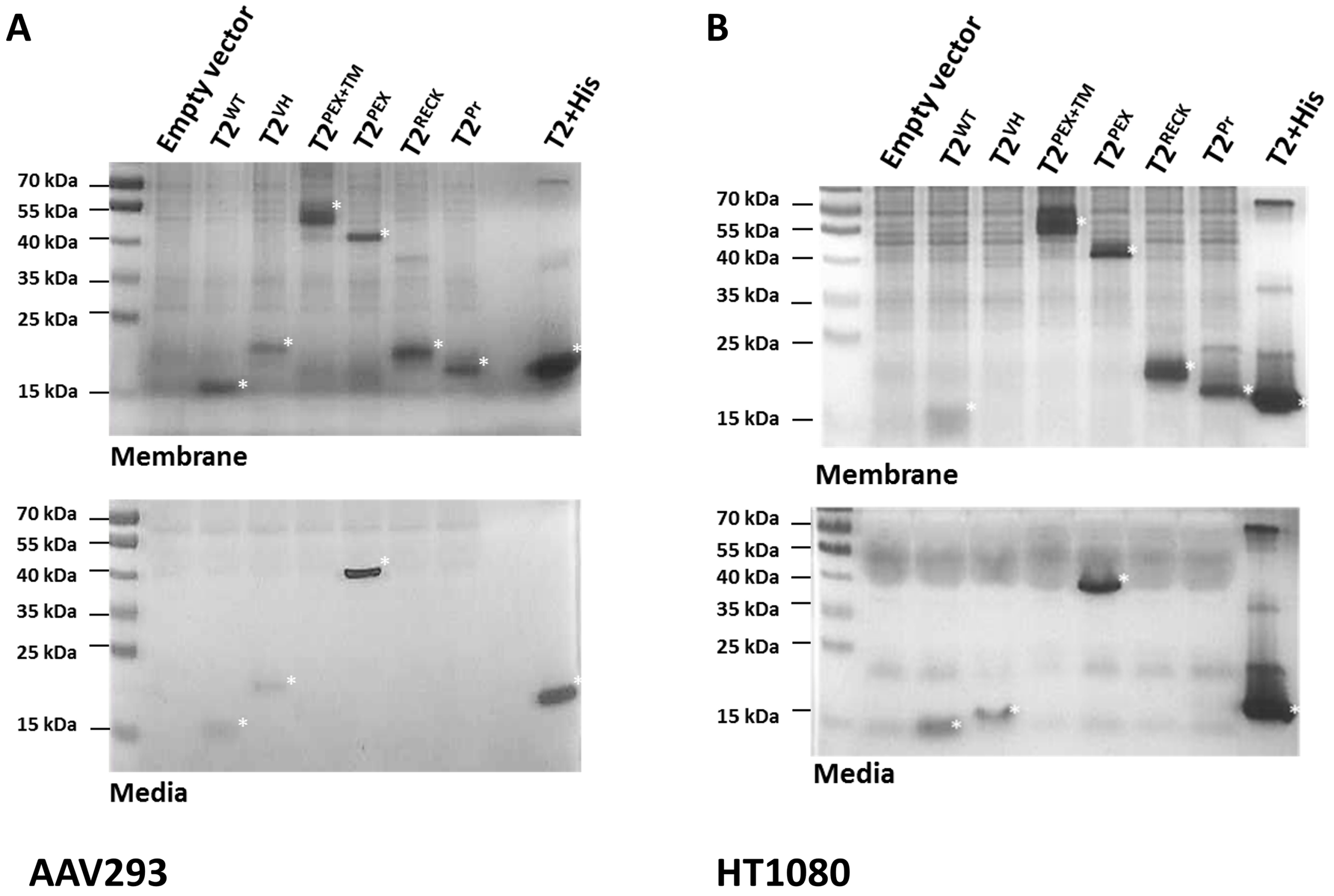

Supplementary Figure 1: Reverse zymography showing the sequestration pattern of membrane-anchored TIMP-2s in transiently-transfected AAV293 and stably-transduced HT1080 cells. (**A**) Reverse zymography showing sequestration of TIMP-2 to the membrane fraction by the C-terminal carriers PEX+TM, PEX, RECK and Pr in transiently-transfected AAV293 cells. (**B**) A similar pattern of distribution was observed in HT1080 cells stably-transduced with lentivirus carrying the TIMP-2 cDNAs. T2+His: purified TIMP-2 with a C-terminal 6x histidine tag as positive control.
